# Exploring Attention-Deficit/Hyperactivity Disorder Symptoms in Patients With Atopic Dermatitis by Disease Severity: Cross-Sectional Analysis

**DOI:** 10.2196/74126

**Published:** 2025-07-15

**Authors:** Amr Molla, Raed Jannadi, Dareen Hafez, Lujain Alrohaily, Ebtesam Abdullah, Duha Azouni, Muayad Albadrani

**Affiliations:** 1 Department of Medicine, College of Medicine, Taibah University, P.O.Box 344, Unit 99, Madinah, 42319, Saudi Arabia, 996 0504342992; 2 Department of Dermatology, Saudi German Hospital, Madinah, Saudi Arabia; 3 Department of Family and Community Medicine and Medical Education, College of Medicine, Taibah University, Madinah, Saudi Arabia; 4 Department of Child & Adolescent Psychiatry, King Salman Medical City, Madinah, Saudi Arabia; 5 Department of Pediatric, King Salman Medical City, Madinah, Saudi Arabia; 6 Department of Medicine, King Fahad Hospital, Madinah, Saudi Arabia

**Keywords:** atopic dermatitis, ADHD, cross-sectional study, neurodevelopmental disorders, attention-deficit/hyperactivity disorder

## Abstract

**Background:**

Atopic dermatitis (AD) is a chronic inflammatory skin condition affecting a significant percentage of the global population. Emerging research suggests a potential link between AD and neurodevelopmental disorders like attention-deficit/hyperactivity disorder (ADHD). However, there is a lack of comprehensive studies within the Saudi Arabian population examining this association.

**Objective:**

This study aims to determine the prevalence of ADHD among patients with AD in Saudi Arabia and to explore potential associations with demographic and clinical factors.

**Methods:**

In this cross-sectional, multicenter study conducted between May and November 2024, 419 patients with AD were recruited from various hospitals in Saudi Arabia. Children were screened for ADHD symptoms using the ADHD Rating Scale-5, while adults were assessed with the Adult Self-Report Scale. Logistic regression was used to evaluate the influence of AD severity, age, gender, nationality, and BMI on the likelihood of ADHD symptoms.

**Results:**

A total of 419 patients with AD were included, of whom 234 (55.8%) were children and 185 (44.2%) were adults; 239 (57%) were female and 360 (85.9%) were Saudi nationals. ADHD symptoms were identified in 84 (20%) patients, with a slightly higher prevalence among children (49/234, 20.9%) compared to adults (35/185, 18.9%; *P*=.61). No significant associations were found between ADHD symptoms and gender, nationality, BMI, or AD severity in either age group. Moderate to severe AD was more common among adults (48/185, 25.9%) than children (42/234, 17.9%; *P*=.048).

**Conclusions:**

This study found that 20% of patients with AD screened positive for ADHD symptoms, with slightly higher rates in children than adults. No significant associations were observed between ADHD symptoms and gender, nationality, BMI, or AD severity. Although no significant clinical predictors were identified, the findings emphasize the need for ADHD screening in patients with AD, particularly in regions with high AD prevalence. Future longitudinal studies should explore underlying mechanisms and assess how managing one condition may influence the other.

## Introduction

Atopic dermatitis (AD) is a chronic, relapsing inflammatory condition of multifactorial origin. It primarily affects infants and children but can persist into adulthood. AD is characterized by chronic itching, following a cycle of flare-ups and remission, typically intensifying at night and showing distinct morphology and distribution based on the patient’s age [1]. Globally, AD affects approximately 5% of the population [2], whereas in Saudi Arabia, it impacts around 13% of individuals [3].

Patients with AD often have a family history of other atopic conditions, including food allergies, asthma, allergic rhinitis, and allergic conjunctivitis. Clinically, AD presents as excoriated, scaly, eczematous papules and plaques, which may become overlaid with bacterial infections, predominantly *Staphylococcus aureus*, leading to yellow crusting and exacerbating the condition [1].

Patients with AD often have comorbid atopic conditions, including asthma and allergic rhinitis, and frequently experience disrupted sleep and psychological stress, which can impact cognitive and emotional regulation [1]. Sleep disturbances due to persistent nocturnal pruritus are particularly problematic and may impair concentration, mood, and behavior, potentially mimicking or exacerbating neurodevelopmental symptoms [4]. Furthermore, the chronic nature of AD can negatively affect quality of life and increase the risk of psychiatric comorbidities, including symptoms consistent with attention-deficit/hyperactivity disorder (ADHD) [5,6].

Recent research has identified a significant link between AD and an elevated risk of ADHD, along with other mental comorbidities and reduced quality of life [5,7]. However, no sufficient studies correlate this relation among the Saudi population.

ADHD is a neurodevelopmental disorder affecting both children and adults, characterized by inattention, hyperactivity, and impulsivity, which can disrupt daily functioning in areas like school, work, and social relationships [6]. In Saudi Arabia, ADHD affects 12.4% of children and around 4% of adults [8]. It is more prevalent in men and presents in three types: inattentive, hyperactive-impulsive, and combined. The etiology of ADHD is multifactorial, involving genetic, environmental, and developmental factors such as maternal smoking, socioeconomic status, and perinatal complications [9].

According to the Diagnostic and Statistical Manual of Mental Disorders, Fifth Edition, Text Revision criteria, ADHD symptoms must appear before age 12, persist in multiple settings, and cause functional impairment. Diagnosis relies on clinical assessment and validated psychometric tools [6,9]. However, ADHD management depends on symptom severity. Mild cases often require psychotherapy, while moderate to severe cases are managed with both pharmacotherapy and psychotherapy. Pharmacotherapy, typically using stimulant and nonstimulant medications, is the first-line treatment [9].

Despite evidence linking AD and ADHD, data in the Saudi population are limited. This study aims to assess the prevalence of ADHD symptoms among adult and pediatric patients with AD in Saudi Arabia and to examine whether AD severity is associated with differences in ADHD symptoms.

## Methods

### Study Setting and Sampling

This cross-sectional, multicenter study aimed to investigate the relationship between ADHD and AD among the Saudi population. A total of 419 adult and child patients with AD were selected to participate in the study by simple random sampling. This research was conducted from May 2024 to November 2024 at participating hospitals across Saudi Arabia. The inclusion criteria include Saudi and non-Saudi male and female patients of various ages diagnosed by a dermatologist with AD, who presented at the participating hospitals and agreed to participate in the study. The exclusion criteria include children younger than 4 years of age, patients whose ADHD symptoms occur exclusively during the course of schizophrenia or another psychotic disorder, and patients with ADHD symptoms that can be better explained by other mental disorders, such as mood disorders. These criteria are outlined in the Diagnostic and Statistical Manual of Mental Disorders, Fifth Edition, making accurate screening in younger children clinically inappropriate.

### Data Collection

Patients with AD with a known diagnosis by a dermatologist, as confirmed from their medical records, were included in the study. Participants were prospectively interviewed and evaluated by a psychiatrist to diagnose ADHD. The ADHD Rating Scale-5 (ADHD-RS-5)-Short Form was used for children, while the Adult Self-Report Scale (ASRS) screening tool was applied for adults. Additional data, including BMI and nationality, were also extracted from medical records to complement the ADHD assessment data. The ADHD screening assessments were administered in both Arabic and English, depending on participant preference and language proficiency. Validated Arabic versions of the ADHD-RS-5 and ASRS tools were used where appropriate.

### Study Variables

#### AD Severity

Diagnoses of AD were confirmed based on historical medical records where the Hanifin and Rajka criteria, one of the earliest and most widely used standards for diagnosing AD, were used by dermatologists [10]. Additionally, these records included Scoring AD (SCORAD) assessments in English, a validated and reliable measure for evaluating AD severity. SCORAD criteria account for extent, intensity, and subjective symptoms, with total scores ranging from 0 to 103. Based on these scores, AD severity is classified into three categories: mild (SCORAD<25), moderate (25≤SCORAD≤50), and severe (SCORAD>50) [11].

#### ADHD

The ADHD-RS-5-Short Form is a simplified screening tool to identify the likelihood of ADHD in children based on parent or teacher observations [12].

##### Structure of the ADHD-RS-5-Short Form

The tool consists of 9 items, each representing a core ADHD behavior. Each behavior is scored on a 4-point scale based on frequency: 0=Never or Rarely, 1=Sometimes, 2=Often, and 3=Very Often.

##### Scoring and Interpretation

The sum of the scores from these 9 items ranges from 0 to 27. A total score of 15 or higher typically indicates a high likelihood of ADHD, suggesting further assessment if other indicators are present. This scoring method serves as an initial screening, with a high score warranting a comprehensive evaluation if ADHD is suspected.

For adults, part A of the Adult Self-Report Scale (ASRS-v1.1) was used, which comprises 6 questions. The first 4 questions assess inattentive (ADHD-I) symptoms, and the last 2 assess hyperactive (ADHD-H) symptoms. Responses range from 0=never to 4=very often. Patients who selected shaded boxes for 4 or more questions were considered to have symptoms consistent with ADHD, indicating a positive screening result [13]. The ASRS-v1.1 has demonstrated strong diagnostic performance, with a reported sensitivity of 91.4% and specificity of 96% for identifying adult ADHD cases, making it a reliable screening tool in both clinical and research settings [14].

### Covariates

The covariates in this study included gender, age, nationality, and BMI. Gender was categorized as male or female. Age groups were defined as children (4‐17 y) and adults (18 y and older). Nationality was classified as either Saudi or non-Saudi; due to the small number and heterogeneity of non-Saudi participants, they were grouped together for analysis. BMI was classified into three groups: normal weight (18.5‐24.9 for adults; 15.5‐21.9 for children), underweight (less than 18.5 for adults; less than 15.5 for children), and overweight or obese (25 or more for adults; 22 or above for children).

### Data Analysis

Data were analyzed using SPSS software (version 29; IBM Corp). Bivariate analysis was conducted by applying the chi-square test to examine associations between categorical variables. Logistic regression analysis was used for multiple variable analysis to assess the influence of gender, age, nationality, BMI, and AD severity on ADHD occurrence. A *P* value of less than .05 was considered statistically significant.

### Ethical Considerations

Ethical approval for this study was obtained from the Institutional Review Board of Taibah University, Saudi Arabia (TU-24-014), on May 15, 2024. All procedures were conducted in accordance with the Declaration of Helsinki and relevant national regulations. Written informed consent was obtained from all adult participants. For participants younger than 18 years of age, written informed consent was obtained from their parents or legal guardians. The study objectives, procedures, data privacy protections, and estimated duration were clearly explained to all participants prior to enrollment. Participation was entirely voluntary, and participants were informed of their right to withdraw at any time without penalty. All collected data were anonymized to ensure confidentiality and stored securely with restricted access. No financial compensation was provided to participants for their involvement in this study.

## Results

### Demographic details

The study included 419 patients with AD, of whom 234 (55.8%) were children and 185 (44.2%) were adults. Among all participants, 239 out of 419 (57%) were female and 180 (43%) were male, with no significant gender distribution difference across age groups (*P*=.27). Most participants were Saudi nationals (360/419, 85.9%), while 59 (14.1%) were non-Saudi (*P*=.16). Regarding BMI, 21 (5%) were underweight, 304 (72.6%) had normal weight, and 95 (22.4%) were overweight or obese. These proportions were similar between children and adults (*P*=.37).

ADHD symptoms were likely in 84 out of 419 (20%) patients, with a slightly higher occurrence among children (49/234, 20.9%) than adults (35/185; 18.9%; *P*=.61). ADHD was likely in 84 out of 419 (20%) patients, with a slightly higher occurrence among children (49/234, 20.9%) than adults (35/185, 18.9%; *P*=.61). AD severity also differed significantly by age group. Among children, 192 (82.1%) had mild AD and 42 (17.9%) had moderate to severe AD, while among adults, 137 (74.1%) had mild AD and 48 (25.9%) had moderate to severe AD (*P*=.048; Table 1). While AD severity differences are reported here, they are included to provide clinical context for interpreting ADHD symptom associations, rather than as a primary focus of the study (Table 1).

**Table 1.** Descriptive characteristics of patients with AD^a^ by age groups.

Variables		Age groups			*P* value^b^
		Study population (N=419), n (%)	Children (n=234), n (%)	Adults (n=185), n (%)	
Sex					.27
Male		180 (43)	95 (40.6)	85 (45.9)	
Female		239 (57)	139 (59.4)	100 (54.1)
Nationality					.16
Saudi		360 (85.9)	206 (88)	154 (83.2)	
Non-Saudi		59 (14.1)	28 (12)	31 (16.8)
BMI					.37
Underweight		21 (5)	10 (4.3)	11 (5.9)	
Normal		304 (72.6)	176 (75.2)	128 (69.2)
Overweight or obese		95 (22.4)	48 (2.5)	46 (24.9)
ADHD^c^					.61
Likely		84 (20)	49 (20.9)	35 (18.9)	
Unlikely		335 (80)	185 (79.1)	150 (81.1)
AD severity					.048
Mild		329 (78.5)	192 (82.1)	137 (74.1)	
Moderate to severe		90 (21.5)	42 (17.9)	48 (25.9)

a AD: atopic dermatitis.

b *P* values were calculated using the *χ2* test.

c ADHD: attention-deficit/hyperactivity disorder.

### Association Between ADHD and Demographic and Clinical Variables in Children With AD

Table 2 shows associations between ADHD and study variables among children with AD. Among 95 male children, 23 (24%) were likely to have ADHD, while among 139 female children, 26 (19%) likely had ADHD (*P*=.31). Among Saudi children (n=206), 47 (22.8%) were likely ADHD cases, compared to 2 out of 28 (7%) non-Saudi children (*P*=.06). Regarding BMI, 1 out of 10 (10%) underweight children was likely to have ADHD, compared to 37 out of 176 (21%) normal-weight children and 11 out of 48 (22.9%) overweight or obese children (*P*=.66). Among 192 children with mild AD, 39 (20.3%) were likely ADHD cases, compared to 10 out of 42 (23.8%) children with moderate to severe AD (*P*=.61).

**Table 2.** Association between ADHD^a^ and study variables among children.

Variables		ADHD			*P* value^b^
		Overall children (n=234), n (%)	Likely (n=49), n (%)	Unlikely (n=185), n (%)	
Gender					.31
Male		95 (40.6)	23 (24.2)	72 (75.8)	
Female		139 (59.4)	26 (18.7)	113 (81.3)
Nationality					.06
Saudi		206 (88)	47 (22.8)	159 (77.2)	
Non-Saudi		28 (12)	2 (7.1)	26 (92.9)
BMI					.66
Underweight		10 (4.3)	1 (10)	9 (90)	
Normal		176 (75.2)	37 (21)	139 (79)
Overweight or obese		48 (20.5)	11 (22.9)	37 (77.1)
AD^c^ severity					.61
Mild		192 (82.1)	39 (20.3)	153 (78.7)	
Moderate to severe		42 (17.9)	10 (23.8)	32 (76.2)

a ADHD: attention-deficit/hyperactivity disorder.

b *P* value calculated by using the *χ2* test.

c AD: atopic dermatitis.

### Association Between ADHD and Demographic and Clinical Variables in Adults With AD

Table 3 presents associations between ADHD and study variables among adult patients with AD. Among 85 male adults with AD, 16 (18.8%) were likely ADHD cases, compared to 19 out of 100 (19%) female adults (*P*=.89). Among 154 Saudi adults, 33 (21.4%) were likely ADHD cases, while only 2 out of 31 (6.5%) non-Saudi adults were likely ADHD cases (*P*=.05). Regarding BMI, 4 out of 11 (36.4%) underweight adults were likely to have ADHD, compared to 25 out of 128 (19.5%) normal-weight adults and 6 out of 46 (13%) overweight or obese adults (*P*=.20). Among 137 adults with mild AD, 29 (21.2%) were likely ADHD cases, while 6 out of 48 (12.5%) adults with moderate to severe AD were likely ADHD cases (*P*=.19).

**Table 3.** Association between ADHD^a^ and study variables among adults.

Variables		ADHD			*P* value^b^
		Overall adults (n=185), n (%)	Likely (n=35), n (%)	Unlikely (n=150), n (%)	
Sex					.89
Male		85 (45.9)	16 (18.8)	69 (81.2)	
Female		100 (54.1)	19 (19)	81 (81)
Nationality					.05
Saudi		154 (83.2)	33 (21.4)	121 (78.6)	
Non-Saudi		31 (16.8)	2 (6.5)	29 (93.5)
BMI					.20
Underweight		11 (5.9)	4 (36.4)	7 (63.6)	
Normal		128 (69.2)	25 (19.5)	103 (80.5)
Overweight or obese		46 (24.9)	6 (13)	40 (87)
AD^c^ severity					.19
Mild		137 (74.1)	29 (21.2)	108 (78.8)	
Moderate to severe		48 (25.9)	6 (12.5)	42 (87.5)

a ADHD: attention-deficit/hyperactivity disorder.

b *P* value calculated by using the *χ2* test.

c AD: atopic dermatitis.

### Logistic Regression Analysis of ADHD Risk Factors in Children With AD

A logistic regression model was applied to examine the influence of gender, nationality, BMI, and AD severity on the likelihood of ADHD symptoms development among children with AD. Male children with AD were 32% more likely to develop ADHD compared to female children (odds ratio [OR] 1.32, 95% CI 0.69‐2.54; *P*=.40). Saudi children were 3.45 times more likely than non-Saudi children with AD to develop ADHD (OR 3.45, 95% CI 0.76‐15.7; *P*=.11). Compared to children with normal BMI, underweight children had a lower probability of developing ADHD symptoms (OR 0.53, 95% CI 0.06‐5.00), while overweight or obese children had a higher risk of ADHD diagnosis (OR 1.25, 95% CI 0.57‐2.74; *P*=.58). Furthermore, children with moderate to severe AD were more likely to have ADHD symptoms (OR 1.40, 95% CI 0.6‐3.25) compared to those with mild AD (*P*=.44; Table 4).

**Table 4.** Logistic regression analysis of study variables influencing ADHD^a^ onset among childhood patients with AD^b^.

Variables		aOR^c^ (95% CI)^d^	*P* value
Sex			
Female		1 (reference)	
Male		1.32 (0.69‐2.54)	.40
Nationality			
Non-Saudi		1 (reference)	
Saudi		3.45 (0.76‐15.7)	.11
BMI			
Normal		1 (reference)	
Underweight		0.53 (0.06‐5)	.58
Overweight or obese		1.25 (0.57‐2.74)	.58
AD severity			
Mild		1 (reference)	
Moderate to severe		1.4 (0.6‐3.25)	.44

a ADHD: attention-deficit/hyperactivity disorder.

b AD: atopic dermatitis.

c aOR: adjusted odds ratio.

d aOR was calculated by including age, gender, nationality, BMI, and AD severity.

### Logistic Regression Analysis of ADHD Risk Factors in Adults With AD

Table 5 assesses the effects of gender, nationality, BMI, and AD severity on the development of ADHD symptoms among adult patients with AD. Male and female adults had nearly the same probability of ADHD symptoms (OR 0.97, 95% CI 0.45‐2.10; *P*=.94). Saudi adults with AD were 3.96 times more likely to have ADHD than non-Saudi adults (OR 3.96, 95% CI 0.89‐17.7; *P*=.07). Compared to adult patients with AD with normal weight, underweight adults with AD had a higher chance of developing ADHD symptoms (OR 2.40, 95% CI 0.63‐9.17), while overweight or obese adults had a lower risk (OR 0.58, 95% CI 0.22‐1.56; *P*=.20 and *P*=.28, respectively). Moreover, adults with moderate to severe AD had a lower probability of having ADHD symptoms (OR 0.54, 95% CI 0.2‐1.43) compared to those with mild AD (*P*=.21). However, none of the examined predictors showed a significant association with ADHD symptoms development among children and adult patients with AD.

**Table 5.** Logistic regression analysis of study variables influencing ADHD^a^ onset among adult patients with AD^b^.

Variables		aOR^c^ (95% CI)^d^	*P* value
Sex			
Female		1 (reference)	
Male		0.97 (0.45‐2.1)	.94
Nationality			
Non-Saudi		1 (reference)	
Saudi		3.96 (0.89‐17.7)	.07
BMI			
Normal		1 (reference)	
Underweight		2.4 (0.63‐9.17)	.20
Overweight or obese		0.58 (0.22‐1.56)	.28
AD severity			
Mild		1 (reference)	
Moderate to severe		0.54 (0.2‐1.43)	.21

a ADHD: attention-deficit/hyperactivity disorder.

b AD: atopic dermatitis.

c aOR: adjusted odds ratio.

d aOR was calculated by including age, gender, nationality, BMI, and AD severity.

## Discussion

### Principal Findings

This study aimed to investigate the association between AD and ADHD in the Saudi population. The findings revealed that 20% of patients with AD were likely to have ADHD symptoms, with slightly higher proportions among children (49/234, 20.9%) compared to adults (35/185, 18.9%). Notably, AD severity varied significantly between age groups, with moderate to severe AD observed more frequently in adults (48/185, 25.9%) than in children (42/234, 17.9%). However, logistic regression analyses showed no statistically significant associations between ADHD and gender, nationality, BMI, or AD severity in either age group. These findings align with the study objectives to explore the strength of association between AD and ADHD symptoms while accounting for patient demographics and clinical factors.

This study contributes novel data from the Saudi population, where research on the co-occurrence of AD and ADHD is currently limited. The inclusion of a large, diverse sample and the use of validated tools for assessing both AD severity (SCORAD) and ADHD symptoms (ADHD-RS-5 and ASRS) enhances the reliability of the findings. Additionally, the multicenter design strengthens the study’s representativeness and generalizability within Saudi Arabia.

The potential link between AD and ADHD symptoms has been increasingly explored in recent studies, with multiple hypotheses proposed to explain this association. One leading theory suggests that systemic inflammation plays a pivotal role in both conditions. Chronic immune dysregulation in AD, particularly the overactivation of Th2-mediated pathways and elevated levels of cytokines such as IL-4, IL-13, and TNF-α, has been implicated in neuroinflammation, which may contribute to ADHD pathogenesis. Studies have shown that children with AD exhibit higher levels of circulating inflammatory markers, which could influence neurodevelopmental processes and neurotransmitter regulation, particularly in dopaminergic pathways linked to ADHD symptoms [15,16]. Additionally, dysregulated sleep patterns, commonly observed in AD due to persistent pruritus, could further impact cognitive function, emotional regulation, and attentional control, exacerbating ADHD symptoms [4].

Another plausible explanation involves the gut-brain-skin axis, which has gained increasing attention in recent research. Alterations in the gut microbiome composition have been linked to both AD and ADHD, suggesting that microbial dysbiosis may serve as a shared pathophysiological factor. Studies have reported reduced microbial diversity and an imbalance in short-chain fatty acid-producing bacteria in both AD and ADHD populations, which may lead to increased intestinal permeability, systemic inflammation, and neurodevelopmental disturbances [17-19].

The results of this study are consistent with findings from previous research, including a US-based study that identified a higher prevalence of ADHD among children with eczema, particularly those with moderate to severe cases [20]. Another study found that ADHD symptoms were more common among children with eczema, highlighting potential shared inflammatory or neurological pathways [21]. Similarly, a German study emphasized the association between moderate to severe AD and mental health issues, although it focused primarily on adolescents [22]. Finally, a Mendelian randomization analysis suggested potential bidirectional causal relationships between AD and psychiatric disorders, further supporting the complex interplay between these conditions [23]. These comparisons validate the relevance of our findings while highlighting differences that may be attributed to cultural, genetic, or methodological factors.

While this study provides valuable insights into the Saudi population, its findings may have limited generalizability to other settings due to cultural, environmental, and genetic differences. The notably higher prevalence of AD in Saudi Arabia (13%) compared to the global average (5%) may reflect unique population-specific risk factors [3]. Additionally, the absence of a control group without AD limits our ability to directly compare the prevalence of ADHD symptoms between patients with AD and the general population. The use of validated screening tools rather than formal clinical diagnostic assessments may also result in over- or underestimation of true ADHD prevalence. Furthermore, several key sociodemographic variables—such as income level, employment status, and psychological well-being—were not collected. Their omission limits our ability to examine broader psychosocial influences on ADHD symptom expression in patients with AD. These factors should be addressed in future studies to strengthen interpretability and generalizability.

Future research should focus on prospective longitudinal studies to better establish causal relationships between AD and ADHD, examining how AD progression or treatment interventions influence ADHD symptoms over time. Exploring genetic markers, inflammatory cytokines, and microbiome alterations in affected individuals may also provide deeper mechanistic insights. Furthermore, studies assessing the impact of AD treatment on ADHD symptomatology could help inform integrated management approaches for patients with coexisting dermatological and neurodevelopmental conditions.

### Conclusions

This cross-sectional study provides important insights into the potential association between AD and ADHD in the Saudi population. Our findings indicate that 20% of patients with AD are likely to have ADHD, with a slightly higher prevalence among children than adults. While no significant associations were found between ADHD and demographic or clinical factors such as gender, nationality, BMI, or AD severity, the study underscores the need for heightened clinical awareness of ADHD symptoms in patients with AD. Given that AD prevalence in Saudi Arabia exceeds global averages, these findings highlight the importance of integrating neurodevelopmental screening into dermatological care.

The growing evidence linking AD to neuropsychiatric disorders suggests shared inflammatory, neuroimmune, and sleep-related mechanisms that warrant further exploration. Future longitudinal studies should aim to establish causality, assess the impact of AD treatments on ADHD symptoms, and investigate potential biomarkers that may mediate this relationship. Understanding these interactions could pave the way for personalized treatment strategies that optimize both dermatologic and neurodevelopmental outcomes, ultimately improving the quality of life for affected individuals.

## Abbreviations

AD: atopic dermatitis
ADHD: attention-deficit/hyperactivity disorder
ADHD-RS-5: Attention-Deficit/Hyperactivity Disorder Rating Scale-5
ASRS: Adult Self-Report Scale
OR: odds ratio
SCORAD: Scoring Atopic Dermatitis
